# Information counselling during treatment with ototoxic medications for TB: a call to action

**DOI:** 10.5588/pha.24.0053

**Published:** 2025-03-01

**Authors:** K. Khoza-Shangase, S.M. Arendse

**Affiliations:** Department of Audiology, School of Human and Community Development, University of the Witwatersrand, Johannesburg, South Africa.

**Keywords:** ototoxicity, tuberculosis, bedaquiline, aminoglycosides, audiology, DR-TB, XDR-TB, South Africa

## Abstract

The introduction of bedaquiline (BDQ) has reduced the reliance on ototoxic drugs (e.g., aminoglycosides) for drug-resistant TB (DR-TB). This has significantly reduced ototoxic symptoms, such as tinnitus and dizziness, which often precede irreversible hearing loss. However, aminoglycosides remain essential for some extensively drug-resistant individuals with TB (XDR-TB) who are resistant to BDQ. In South Africa, there is a lack of adequate awareness of the potential ototoxic effects of aminoglycosides. The increasing prevalence of XDR-TB has, therefore, intensified the need for comprehensive information counselling. We call for a proactive role for audiologists in providing early, structured information counselling for these individuals.

South Africa continues to grapple with one of the highest rates of drug-resistant TB (DR-TB), and treatment challenges are constantly evolving.^1,2^ The shift towards the use of bedaquiline (BDQ) instead of injectable aminoglycosides represents a major development with implications for ototoxicity and the role of audiologists.^1,3,4^ Although BDQ presents a less ototoxic option for DR-TB treatment, limitations in its availability mean that aminoglycosides are still widely administered, especially for extensively drug-resistant TB (XDR-TB) strains resistant to bedaquiline.^[4]^ This calls for greater involvement from South African audiologists in providing proactive information counselling to manage the complex implications of ototoxic medications.

## Transitioning to BDQ and the ongoing use of aminoglycosides

WHO has promoted the replacement of injectable aminoglycosides with BDQ due to its significantly reduced risk of ototoxicity.^5^ BDQ has been a breakthrough, especially for multidrug-resistant TB (MDR-TB) and many XDR-TB cases, as it allows for more effective, shorter treatment regimens without the heightened risk of irreversible hearing loss.^3,4,6,7^ Research supports this approach, with studies finding no significant hearing threshold shifts in individuals treated with BDQ compared to the ototoxic effects observed in those receiving aminoglycosides.^3,4,7,8^ However, BDQ resistance is an emerging concern, leaving some persons with XDR-TB reliant on injectable aminoglycosides, which are ototoxic.^9^ Paradoxically, this more limited use of aminoglycosides may increase the risk of ototoxicity, as fewer individuals receiving these drugs could lead to a reduction in counselling and audiometry programmes. For those individuals who must remain on aminoglycosides due to BDQ resistance or contraindications, ototoxicity risk management remains essential. With the limited alternative treatments available, audiologists must be prepared to provide early information counselling to ensure they understand the risks and symptoms of ototoxicity, which includes hearing loss, tinnitus, dizziness and balance issues.^10^

## Information counselling: a foundation for informed decision-making

Comprehensive information counselling is critical to improving a person with XDR-TB's understanding of potential side effects. Counselling helps these individuals recognise early ototoxic symptoms (such as tinnitus and vertigo), which can precede irreversible hearing loss. According to the Health Professions Council of South Africa (HPCSA) guidelines,^11^ persons prescribed ototoxic medications should be counselled prior to starting treatment. However, there are significant inconsistencies in both the timing and quality of this counselling. We led a recent study to investigate awareness of ototoxic effects among adults with XDR-TB undergoing treatment in South Africa, focusing on the role of information counselling, timing of counselling and access to audiological monitoring.^12^ Using a cross-sectional, descriptive qualitative design, semi-structured interviews were conducted with 10 individuals with XDR-TB at Brooklyn Chest Hospital in the Western Cape. Ethical clearance was provided by the University of the Witwatersrand’s Human Research Ethics Committee (Medical) (No. M240420). Consent to participate was obtained from the participants and the relevant hospital authorities as part of the ethics approval process. Thematic analysis revealed significant variability in the content and timing of information counselling. Only 30% of participants reported receiving comprehensive counselling on ototoxic symptoms, whereas 70% received general information lacking specific details about hearing-related risks. Regarding timing, 60% of participants received counselling before treatment initiation, 30% only after treatment had started, and just 10% at both points. Regarding audiological assessments, 40% received a baseline test before treatment, whereas 50% had their baseline assessment only after treatment started, with only one participant receiving follow-up testing. Half of the participants experienced ototoxic symptoms (such as dizziness and tinnitus), yet gaps in both counselling and follow-up assessments highlight a need for improved, standardised practices. These findings highlight the importance of comprehensive, timely counselling and regular audiological monitoring to enhance early symptom recognition and management.^12^ Other studies found that only a small proportion of individuals with XDR-TB received thorough information counselling, and many individuals were unaware of all ototoxic risks associated with aminoglycosides.^13,14^

The effectiveness of pre-treatment information counselling is well-documented. Individuals who understand the nature of their treatment and its side effects are more likely to adhere to prescribed regimens, as they are prepared for possible adverse events and equipped with the knowledge to report early symptoms.^15^ Studies in South Africa have shown that those who receive timely, comprehensive counselling report feeling more empowered, which positively influences their engagement with treatment.^3,16–18^ By providing accurate, culturally relevant counselling, audiologists can support individuals in making informed decisions about their care, especially in rural or underserved areas where healthcare resources are scarce.

## The role of audiologists

Audiologists are uniquely positioned to offer preventive care through information counselling and must, therefore, engage directly with persons with TB to ensure they are informed of potential hearing risks. This aligns with the WHO’s call for a multidisciplinary, person-centred approach to TB treatment, particularly for high-burden settings like South Africa.^19^ However, for audiologists to fulfil this expanded role effectively, systemic changes are necessary. Most public hospitals in South Africa do not have robust ototoxicity monitoring programmes in place, resulting in delayed diagnosis and intervention for individuals experiencing auditory side effects.^20^ Regular follow-up is crucial, especially as symptoms like tinnitus or dizziness may develop gradually, and individuals may not recognise them as signs of potential hearing loss.

## Challenges and the need for systemic support

Implementing routine counselling and consistent follow-up for persons on ototoxic medications presents significant challenges in South Africa’s public health system. Limited resources, heavy caseloads, and insufficient training in ototoxicity monitoring are common obstacles.^20,21^ Despite the recognised need for baseline hearing assessments within 72 hours of treatment initiation, the reality is that many individuals do not receive timely evaluations, and follow-up testing is often inconsistent.^15,16,22^ To address these issues, health policies should prioritise ototoxicity monitoring and audiological counselling as standard components of TB care. This will require investment in both human and technical resources and training for audiologists and cognate healthcare team members, including community healthcare workers, to provide culturally competent care tailored to the diverse patient population in South Africa. By integrating audiologists into the broader treatment team, healthcare providers can help ensure that all persons with TB – regardless of their treatment regimen – receive the necessary support and monitoring to protect their auditory health.

## Fostering culturally sensitive counselling

Effective counselling must be culturally sensitive, as language and cultural differences significantly impact how patients perceive health information.^23^ For example, information counselling should empower patients to report symptoms autonomously, yet studies show that linguistic barriers often inhibit this empowerment.^24–26^ In cases where patients are unaware of the symptoms to report or are unclear about whom to consult, early symptoms may go unnoticed, and opportunities for intervention are lost. To improve patient outcomes, audiologists must prioritise the delivery of informational materials in multiple languages, adapting communication styles to suit patients’ needs, and involving family members in counselling sessions where appropriate. Given that many individuals with TB may experience economic or social barriers to accessing regular healthcare, audiologists should also be trained to engage in clear, accessible communication that promotes understanding and active participation in monitoring their symptoms.

## A CALL TO ACTION

Based on these challenges, we make the following recommendations and urge healthcare policymakers, providers and public health stakeholders to implement these changes. This call to action emphasises the need for standardised counselling protocols, enhanced accessibility to audiological care, and integrated support services to ensure that individuals with XDR-TB are adequately informed, monitored and supported throughout their treatment journey.

1 *Establish standardised protocols for ototoxicity counselling.* South African healthcare systems must develop protocols for audiologists to ensure consistent and comprehensive information counselling for all individuals on ototoxic medications. This includes training in effective communication strategies, cultural competence, and tailored approaches based on individual patient needs and treatment regimens.
2 *Increase access to ototoxicity monitoring programmes.* South Africa should expand access to community-based ototoxicity monitoring, particularly in rural areas where healthcare access is limited, with serious planning around task shifting as there is a well-documented capacity versus demand challenge as far as audiologists are concerned.
3 *Integrate tele-audiology and mobile services.* Expanding tele-audiology services can increase access to essential hearing assessments and follow-up appointments, allowing audiologists to provide continuous care without overburdening hospital resources. Mobile health services can similarly bridge gaps in care for persons unable to travel to centralised facilities.
4 *Strengthen collaboration within multidisciplinary teams.* Audiologists must be integral members of multidisciplinary TB treatment teams, contributing their expertise in ototoxicity management to support person-centred care. Closer collaboration with TB physicians, nurses, and pharmacists can ensure patients receive holistic support that considers both the efficacy and side effects of their treatment.
5 *Conduct longitudinal research on BDQ and aminoglycosides.* Ongoing research into the auditory effects of BDQ and aminoglycosides will provide critical insights into individual experiences and inform best practices for information counselling. Studies should aim to identify patterns in symptom onset, personal awareness, and factors that impact early reporting, particularly in high-risk groups.

## CONCLUSION

The shift towards using BDQ for TB treatment is promising, but the continued use of aminoglycosides for XDR-TB cases presents an urgent need for audiologist involvement in information counselling. By providing comprehensive, culturally sensitive counselling, audiologists can empower individuals to engage proactively with their health and recognise ototoxic symptoms early. Audiologists must advocate for the resources and systemic changes needed to make ototoxicity monitoring a routine part of TB care, ensuring all persons with TB have access to the support necessary to prevent avoidable hearing loss.
